# Myoclonus-Dystonia Plus Syndrome With Early-Onset Multiple Cerebral Cavernous Malformation Type 1 and Growth Hormone Deficiency Associated With Novel 7q21.13-q21.3 Deletion: A Pediatric Case Report

**DOI:** 10.7759/cureus.56294

**Published:** 2024-03-16

**Authors:** Kohei Matsubara, Ichiro Kuki, Yuki Yamada, Jun Mori, Shin Okazaki

**Affiliations:** 1 Pediatric Neurology, Osaka City General Hospital, Osaka, JPN; 2 Pediatric Endocrinology and Metabolism, Osaka City General Hospital, Osaka, JPN

**Keywords:** krit1, growth hormone deficiency, cerebral cavernous malformation, sgce, myoclonus-dystonia syndrome, 7q21 deletion

## Abstract

Myoclonus-dystonia syndrome (MDS) presents with both rapid myoclonus and dystonia, which is caused by mutations in the sarcoglycan (SGCE) gene. However, its complications and management remain unclear. Here, we report a case involving a girl with MDS due to a 7q21.13-q21.3 microdeletion complicated by early-onset multiple cerebral cavernous malformations (CCMs). The patient presented with myoclonus and dystonia at two and eight years of age, respectively. In addition to MDS, the patient developed growth hormone (GH) deficiency and mild intellectual disability. Magnetic resonance imaging of the brain showed multiple CCMs. Array-based comparative genomic hybridization revealed 7q21.13-21.3 microdeletion. The deletion size was 4.11 Mb, which included SCGE and KRIT1. After the introduction of zonisamide, both myoclonus and dystonia showed improvement, and GH therapy led to an increase in patient height. In cases of MDS, multiple early-onset CCMs and GH deficiency may occur; moreover, careful follow-up management may be necessary.

## Introduction

Myoclonus-dystonia syndrome (MDS) presents with both rapid myoclonus, known as myoclonic jerks, and dystonia beginning in childhood [1]. Myoclonus appears more often in the neck and upper extremities than in the trunk, while dystonia appears as torticollis or chirospasms, with symptoms often milder than those of myoclonus. Based on an analysis of a large cohort with essential familial myoclonus-dystonia, Nygaard et al. [2] reported that the gene responsible for MDS was located on 7q21-q31. Furthermore, Zimprich et al. [3] reported that mutations in the ε-sarcoglycan (SGCE) gene, which is located at 7q21.3, were the cause. SGCE is expressed in several brain regions, such as the cerebral cortex, cerebellum, hippocampus, and striatum [4]. Loss of SGCE induces neuronal membrane damage, leading to calcium accumulation and dysfunction of the dopamine D2 receptor, which is caused by myoclonus and dystonia [4].

Cerebral cavernous malformation (CCM) is a vascular malformation of the central nervous system that occurs mainly in adulthood and is associated with headaches, vomiting, and seizures. CCM1, CCM2, and CCM3 have been reported to be the genes responsible for CCM. CCM1, also known as KRIT1, is located on 7q21.2 [5].

In this report, we describe a case involving a young girl with MDS due to a 7q21.13-q21.3 microdeletion complicated by early-onset multiple CCM.

## Case presentation

**Figure 1:**
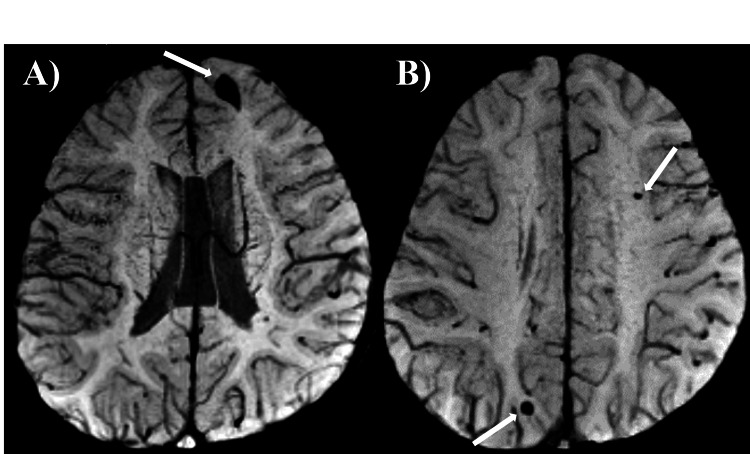
Brain magnetic resonance imaging at six years of age (A, B) Multiple small hypointense lesions in the left frontal lobe (A), left parietal lobe and right occipital lobe (B) were detected on T2*-weighted magnetic resonance imaging.

**Figure 2:**
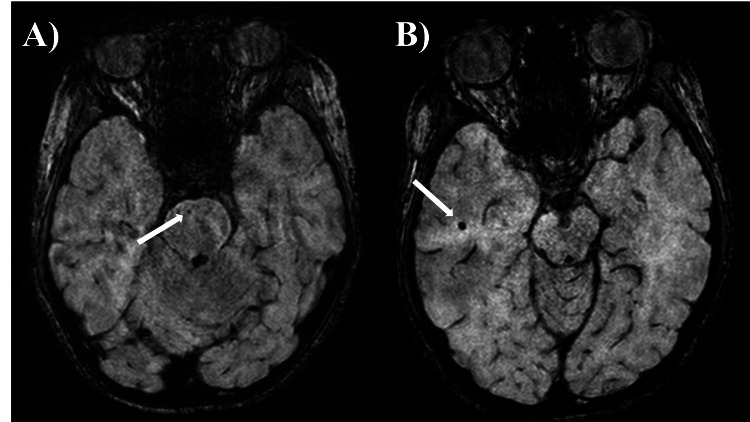
Brain magnetic resonance imaging at the 11 and 13 years of age The appearance of new lesions in the pons and right temporal lobe was confirmed on susceptibility-weighted imaging.

**Figure 3:**
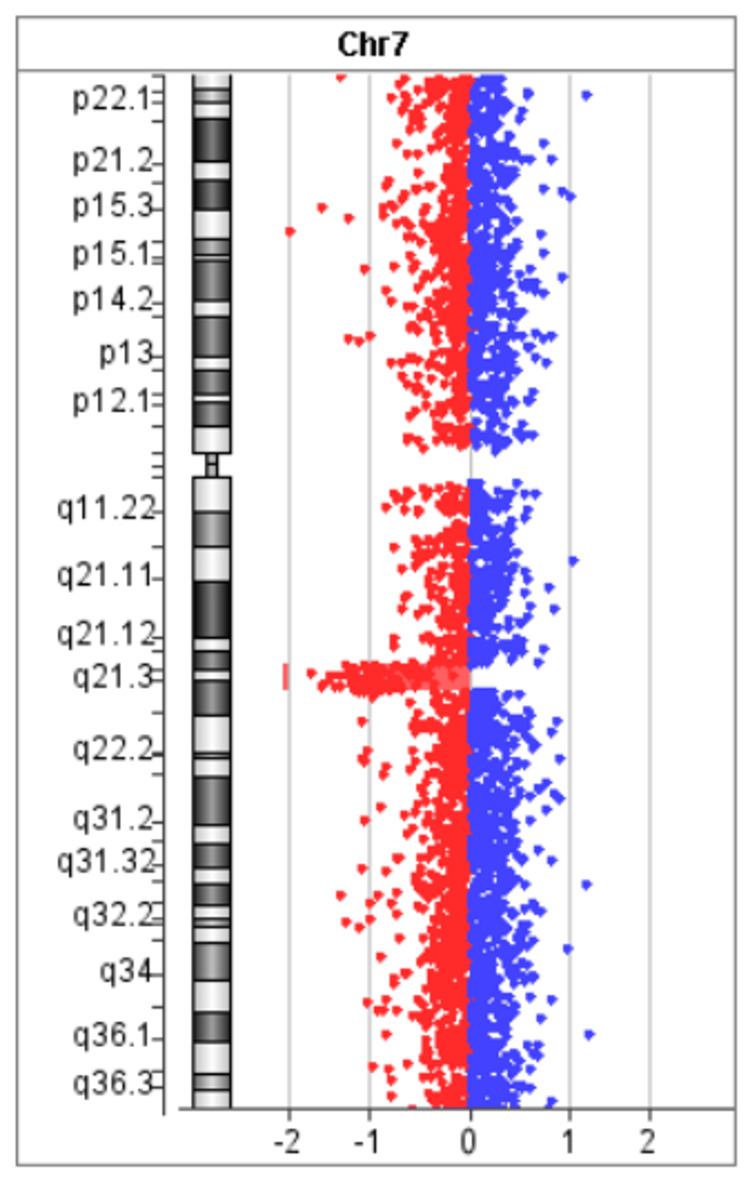
Array-based comparative genomic hybridization The figure shows a 7q21.13-q21.3 deletion in a 14-year-old girl with myoclonus–dystonia plus syndrome and multiple early-onset type-1 cerebral cavernous malformation, with a deletion extent of 4.11 Mb.

The patient was a 14-year-old girl. There was no prenatal laboratory testing, and she was born vaginally without asphyxiation or neonatal intensive care unit admission. There were no genetic conditions or syndromes in her family, including myoclonus or dystonia. She began to experience rapid, uncontrollable movements of both the upper limbs and neck from two years of age. She was referred to our hospital for a short stature at four years of age and to our division for investigation of myoclonus at five years of age. At the initial visit, her height was 98 cm (-2.5 SD), and her weight was 12.8 kg. She had neither dysmorphic features other than bilateral ear fistula, nor cranial nerve abnormalities, muscle weakness, muscle tonus, pathological reflexes, and Romberg's sign. She presented with quick movements of both the upper extremities and the neck without electroencephalographic changes and was diagnosed as having myoclonus. Blood and cerebrospinal fluid tests revealed no abnormalities. Electrocardiography revealed a sinus rhythm and no QT prolongation. The results of chromosome G staining were 46, XX. Head magnetic resonance imaging (MRI) showed low-signal lesions on T2*-weighted sequence in the left frontal lobe, left parietal lobe, and right occipital lobe, suggesting multiple CCM (Figure 1). She was started on oral clonazepam for myoclonus; however, her symptoms did not improve. At eight years of age, the patient developed dystonia and was clinically diagnosed with MDS. After the introduction of zonisamide, both myoclonus and dystonia were improved. She was diagnosed as having a mild mental impairment with an intelligence quotient of 77 on the Wechsler Intelligence Scale for Children, Fourth Edition, at nine years of age. There were no endocrinological abnormalities except for a low insulin-like growth factor 1 level of 49 ng/ml. A growth hormone (GH) stimulation test revealed that the peak GH values were 3.76 and 5.41 ng/ml after arginine and L-Dopa administration, respectively. The patient was diagnosed with GH deficiency at nine years of age. GH therapy was started, and her height increased. SGCE analysis at age 12 revealed no pathological variants, but a hetero-large deletion was suspected. The array-based comparative genomic hybridization revealed 7q21.13-21.3 microdeletion, and the extent of deletion was found to be 4.11 Mb (Figure 2). The breakpoint was at chr7:90,366,644-94,478,062. Although she had no neurological symptoms, head susceptibility-weighted imaging MRI showed a low-signal lesion in the pons and right temporal lobe at 11 and 14 years of age, respectively (Figure 3). At the last evaluation (age 14 years), her height was 148 cm (-1.6 SD).

## Discussion

This is a pediatric case of MDS presenting with early-onset multiple CCM and GH deficiency. Although multiple CCMs have been reported in adult patients with MDS associated with 7q21 microdeletion, there have been no reports in pediatric patients. In this case, the deletion region 7q21.13-q21.3 was considered to be the cause of the disease, which contained both SGCE and KRIT1 genes, the cause of MDS and familial CCM, respectively.

DeBerardinis et al. [6] reported a pediatric case of deletion of 7q21, which was associated with microcephaly, short stature, language developmental delay, and mild facial malformations in addition to myoclonus, suggesting that symptoms may reflect the gene mutation located in the deletion region. Courtens et al. [9] reported a boy with a 7q21.1-q21.3 deletion who presented with moderate developmental delay, mild facial deformity, and short stature without myoclonus and dystonia, suggesting that the deletion region may not contain SGCE. Asmus et al. [7] summarized the clinical characteristics of four patients (one of whom was previously reported by DeBerardinis) with a deletion in the 7q21.3 region and reported that the size of the genomic deletion at the SGCE locus determined the clinical phenotype. Kim et al. [10] reported a case of multiple malformations involving split hand-and-foot malformation with a 7q21.13q22.1 deletion. The report emphasizes that high-resolution cytogenetic techniques have enabled the identification of more detailed breakpoints, the subsequent re-establishment of phenotype-genotype correlations, and the discovery of genes related to specific phenotypes.

Regarding CCM, Tzschach et al. [11] reported an adult case with a 7q21.1-q21.3 deletion that showed a single CCM in the left frontal lobe, the first CCM case associated with a 7q21 deletion. In the report by Asmus et al. [7], deletion of 7q21.2, where KRIT1 is located, was noted in three patients (two pediatric and one adult). However, only the adult patient presented with CCM. This can be attributed to the fact that the average age of onset of KRIT1-related CCM is 30 years [12]. In addition, Labauge et al. [13] proposed the hypothesis that a second somatic mutation is required for the onset of CCM when a heterozygous mutation in KRIT1 is present. However, in our case, multiple CCMs were already present at four years of age, which is inconsistent with the previously reported clinical course. As in previous reports, no symptoms were observed; however, the appearance of new lesions suggests that careful follow-up is necessary. Although the reason why CCMs developed earlier in our case is not unclear, non-genetic factors may be involved because patients with the same microdeletion as in our case did not present with early-onset CCMs.

**Figure 4:**
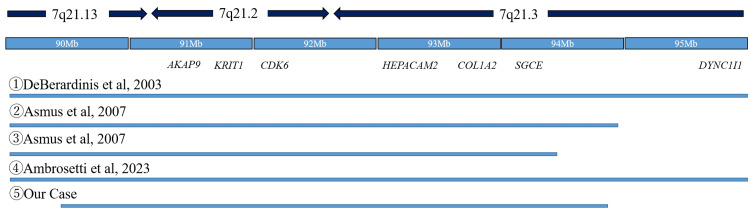
A summary of cases of 7q21 microdeletions, including SGCE and KRIT1 genes This figure shows the extent of deletion comparing the present case with previous reports. DeBerardinis et al. [6] reported a pediatric case of deletion of 7q21. Asmus et al. [7] reported the clinical characteristics of two new patients with a deletion in the 7q21.3 region including SGCE and KRIT1. Ambrosetti et al. [8] reported a pediatric patient of 7q21 microdeletion with split hand-foot and deafness.

Short stature is often observed in 7q21 deletion cases [6-9, 11], and Asmus et al. [7] reported that blue sclerae/joint laxity, hypodontia, bone fracture, and healing loss, including short stature, are caused by haploinsufficiency of COL1A2 located in the deletion region. Interestingly, however, the case reported by Courtens et al. [9] showed short stature despite the absence of COL1A2 in the deletion region. In general, osteogenesis imperfecta type I caused by COL1A2 haploinsufficiency has a normal GH axis; [14] however, the patient in this study presented with GH deficiency, and GH treatment resulted in an increase in height. Although there have been no reports of the GH deficiency phenotype and GH treatment, the treatment was effective in this case. These results suggest that the short stature in 7q21 deletion cases may not be solely a component of COL1A2 haploinsufficiency. AKAP9, the causative gene of Long QT syndrome type 11, is located at 7q21.2; however, no QT prolongation was observed in this case. Although it is generally known that MDS patients with SGCE mutations do not show cognitive dysfunction [15], our patient showed mild developmental delay. CDK6 and HEPACAM2 have been postulated as the cause of intellectual disability in MDS patients with a background of 7q21 microdeletion [16]. Because these genes were also included in the deletion region in this case, they may be the cause of intellectual disability. In a recent study, Ambrosetti et al. [8] reported a case of 7q21.13-q21.3 deletion with split hand-and-foot malformation and deafness. The deletion region did not contain DLX5/6, the cause of SHFM1, and DYNC1I1 proved to be the cause of the symptoms in this case. This case also contained SGCE in the deletion region but did not present myoclonus dystonia. Cases of 7q21 microdeletion can be complicated by various symptoms in addition to MDS, as reported previously, and the disease concept of MDS has been proposed [7, 16]. Because this patient had early-onset multiple CCM, GHD, and mild intellectual disability in addition to MDS, we diagnosed her with MDS. We also summarized previously reported cases that were similar to the present case (Table 1, Figure 4).

**Table 1:** A summary of cases of 7q21 microdeletions, including SGCE and KRIT1 genes This table summarized the case of 7q21 microdeletions, including SGCE and KRIT1 MDS - myoclonus-dystonia syndrome; CCM - cerebral cavernous malformation; NA - not applicable

Case	Age	Sex	Deletion area	Deletion size	MDS	CCM	Short stature	Intellectual disability	Long QT syndrome	Other features
DeBerardinis et al. [6]	2 years	male	7q21.11-21.3	15.5Mb	+	-	+	+	N/A	Ffacial dysmorphism
Asmus et al. [7]	59 years	female	7q21.13-21.3	4.99Mb	+	+	+	-	N/A	Blue scrlae, joint laxity
Asmus et al. [7]	9 years	male	7q21.13-21.3	8.78Mb	-	-	+	+	N/A	Joint laxity, hypodontia, Mondini dysplasia
Ambrosetti et al. [8]	9 years	male	7q21.13-21.3	6.29Mb	-	-	+	-	N/A	Split hand-foot, deafness
Our case	14 years	female	7q21.13-21.3	4.11Mb	+	+	+	+	-	Bilateral ear fistula

Benzodiazepine receptor agonists, such as clonazepam, which reduces the excitability of GABAergic neurons, are used in MDS with SGCE mutations. However, the effect was limited, with only mild or no improvement, and the symptoms did not improve in this case. Zonisamide is the only drug proven to be effective in MDS patients with SGCE mutations in a double-blind, randomized controlled trial [17], but it has not been reported for use in MDS with 7q21 microdeletions; however, it was effective in this case.

This report has two limitations. First, neither the parents nor the older brother underwent array-based comparative genomic hybridization testing; thus, we could not confirm whether the microdeletion observed in this case was de novo. Second, we could not identify any cases of short stature related to genetic abnormalities located at 7q21 as far as we investigated; hence, we could not discuss this point.

## Conclusions

Our case of 7q21.12-21.3 microdeletion was considered to be an MDS because it was associated with various symptoms, such as CCM, short stature associated with GH deficiency, and mild developmental delay in addition to MDS. Although there have been previous cases of adult-onset CCM in MDS, this case showed early-onset multiple CCM. While no hemorrhagic symptoms, such as headache or vomiting, were observed, multiple CCMs were present in the bilateral cerebral hemispheres, pons, and cerebellum, necessitating careful follow-up.
